# Inflammatory and Angiogenic Mediators Are Differentially Ex-Pressed in Patients with Post-COVID-19 Syndrome with Normal and Abnormal Spirometry Results

**DOI:** 10.3390/healthcare13111346

**Published:** 2025-06-05

**Authors:** Laura Ileana Minjarez-Robles, Jesús Gilberto Arámburo-Gálvez, Oscar Gerardo Figueroa-Salcido, José Manuel Ornelas-Aguirre, Noé Ontiveros, Lilian Karem Flores-Mendoza

**Affiliations:** 1 Health Sciences Postgraduate Program, Department of Chemical, Biological, and Agricultural Sciences (DC-QB), Faculty of Biological and Health Sciences, University of Sonora, Navojoa 85880, Sonora, Mexico; laura.minjarezr@gmail.com; 2 Family Medicine Unit, Number 1, National Northeast Medical Center, Mexican Social Security Institute, Ciudad Obregon 85130, Sonora, Mexico; 3 Nutrition Sciences Postgraduate Program, Faculty of Nutrition and Gastronomy Sciences, Autonomous University of Sinaloa, Culiacan 80019, Sinaloa, Mexico; gilberto.aramburo@uas.edu.mx (J.G.A.-G.); oscar.figueroa@uas.edu.mx (O.G.F.-S.); 4 Integral Postgraduate Program in Biotechnology, Faculty of Chemical and Biological Sciences, Autonomous University of Sinaloa, Ciudad Universitaria, Culiacan 80010, Sinaloa, Mexico; 5 Specialties Hospital Number 2, Sub-Specialty Medical Unit, National Northeast Medical Center, Mexican Social Security Institute, Ciudad Obregon 85130, Sonora, Mexico; manuel.ornelas@unison.mx; 6 Clinical and Research Laboratory (LACIUS, C.N., CONAHCYT National Laboratory, LANIBIOC), Department of Chemical, Biological, and Agricultural Sciences (DC-QB), Faculty of Biological and Health Sciences, University of Sonora, Navojoa 85880, Sonora, Mexico

**Keywords:** SARS-CoV-2, cytokine, inflammation, post-COVID syndrome

## Abstract

Background: Inflammatory and angiogenic mediators play a key role in post-COVID-19 syndrome pathophysiology. These mediators might be of prognostic value for pulmonary function in this syndrome. Objectives: To determine interleukin-6, -12, and -17, macrophage inflammatory protein-1A (MIP-1A), the vascular endothelial growth factor-A (VEGF-A) gene expression levels, the matrix metalloproteinase-9 (MMP-9) plasma levels, and the association of clinical data with pulmonary function in patients with post-COVID-19 syndrome with normal and abnormal spirometry results. Methods: Demographic/clinical data and blood samples were collected (45 patients). Pulmonary function was evaluated (spirometry), and the gene expression levels of inflammatory and angiogenic mediators (IL-6, IL-12, IL-17, MIP-1A, and VEGF-A) were determined in PBMCs (qPCR). MMP-9 plasma levels were determined (ELISA). Results: Seventeen out of forty-five patients with post-COVID-19 syndrome had abnormal spirometry values, which were associated with arterial hypertension, pneumonia, previous hospitalization, and disease severity (*p* < 0.05). IL-6, IL-12, and VEGF-A gene expression was upregulated in patients with post-COVID-19 syndrome compared with healthy controls. In patients with normal spirometry values, IL-17 and VEGF-A gene expression was upregulated (*p* < 0.05), but MIP-1A was downregulated (*p* < 0.05) (vs. the abnormal spirometry group). MMP-9 serum levels were increased in the normal spirometry group compared with the abnormal one (*p* < 0.05). Conclusions: Post-COVID-19 syndrome has a complex immune pathophysiology, but potential inflammatory and angiogenic biomarkers, such as IL-6, IL-12, IL-17, MIP-1A, and VEGF-A, are differentially expressed in this syndrome and might be prognostic predictors of post-COVID-19 syndrome associated with pulmonary function alterations.

## 1. Introduction

Post-COVID-19 syndrome (also known as long COVID) is a potentially disabling disease defined as the persistence of COVID-19-related symptoms for more than three months after being infected with sudden acute respiratory syndrome-coronavirus-2 (SARS-CoV-2) [1]. In fact, between 17% and 62% of patients with post-COVID-19 syndrome have serious pulmonary sequelae, affecting their quality of life, physical and emotional well-being and their ability to work [2]. The worldwide incidence of the syndrome varies between 10% and 35% [1], but it reaches up to 85% in hospitalized COVID-19 patients [3,4]. The most frequent symptoms in patients with post-COVID-19 syndrome are fatigue, dyspnea, cough, and thoracic pain, but in other cases the syndrome involves myalgias, olfactory and taste disturbances and even mental and sleep disorders that can occur in the absence of the most common manifestations [5,6]. Interestingly, both Intensive Care Unit (ICU) patients admitted due to severe manifestations and those with mild or moderate symptoms of acute COVID-19 can develop post-COVID-19 syndrome [7].

Although SARS-CoV-2 infection per se damages the respiratory epithelium and the vascular endothelium [8], the patient’s immune response, which involves an increased migration of monocytes and neutrophils to pulmonary tissue, contributes at greater extent to the vascular endothelial damage [9]. The recruitment of immune cells promotes the production of leukotrienes and reactive oxygen species (ROS), among other immune mediators, causing plasma leakage into the alveolar space and increasing the procoagulant state [9]. Notably, the cytokines (tumor necrosis factor-alpha (TNF-α), interleukin-1beta (IL-1β), IL-6, IL-8, IL-12, and IL-17, macrophage inflammatory protein (MIP-1), vascular endothelial growth factor (VEGF)), chemokines (CXCL1 and CXCL8), ROS, and metalloproteinases 1 and 9 (MMP-1 and MMP-9) triggered in the acute phase of COVID-19 can promote alveolar destruction and tissue remodeling, leading to pulmonary fibrosis [2,10,11,12]. Certainly, post-COVID-19 syndrome can develop irrespective of the intensity of the symptoms triggered in the acute phase of SARS-CoV-2 infection and/or the presence or absent of respiratory manifestations [13,14]. The characteristics of this illness draw attention to determination of immune and angiogenic mediators that could contribute to our understanding of post-COVID-19 syndrome development. Furthermore, it can be hypothesized that inflammatory and angiogenic molecules are differentially expressed in patients with post-COVID-19 syndrome with and without respiratory complications, and their determination might be of prognostic value for pulmonary function in this syndrome. Thus, in the present study, the plasma levels of MMP-9 and the gene expression of IL-6, IL-12, IL-17, VEGF-A, and MIP-1A in the peripheral blood mononuclear cells of patients with post-COVID-19 syndrome with normal and abnormal spirometry results were determined.

## 2. Materials and Methods

### 2.1. Ethical Statement

The present study was conducted according to international ethical principles and the Declaration of Helsinki. An Ethics and Research Committee of the Mexican Social Security Institute [Specialties Hospital Number 2, in Ciudad Obregon, Sonora, Mexico] approved the research protocol [registration number: R-2022-2602-044]. All patients were informed of the nature of this study and signed a letter of informed consent. A code number was assigned to participants to assure their confidentiality.

### 2.2. Evaluation of the Recruitment Instrument and Participants Selection

A 17-question instrument was designed to collect data about epidemiological characteristics, risk factors, the persistence of clinical symptoms, and comorbidities. Other clinical information was obtained from medical records. Two pulmonologists evaluated the pertinency of the questions. Clarity, comprehension, and readability of the questions were evaluated in a cohort of 50 subjects, who were recruited at a medical unit waiting area in the Specialties Hospital Number 2. Clarity and comprehension of the questions was analyzed using a continuous scale with 3 (1 = clear and comprehensive; 2 = difficult to understand; 3 = incomprehensible) and 10 points (0 = very easy to understand; 10 = very difficult to understand). Average values < 2 (three-point scale) and questions rated ≤ 3 (ten-point scale) were the cut-off values for considering the questions clear and comprehensible [15,16]. The readability of the questions was evaluated using the Flesch–Kincaid test and the score calculated using the INFLESZ software version 1.0 (cut-off value ≥ 60) [17].

Two hundred and thirty-three patients with both SARS-CoV-2 infection confirmed by RT-PCR test and medical diagnosis of post-COVID-19 syndrome were contacted by telephone and asked about the persistence of COVID-19-related symptoms. All these patients were diagnosed in the period from January to October 2022 and were invited to participate in the study (from January to June 2023). Patients with acute SARS-CoV-2 infection at the moment of the study and/or a history of chronic pulmonary illness, cancer, autoimmune diseases, human immunodeficiency virus, sarcoidosis, and/or pulmonary tuberculosis were excluded from this the study. Participants with persistent COVID-19-related symptoms and that agreed to participate in the study were scheduled to attend an appointment. For the purposes of gene expression estimates, this study included a healthy control group (apparently healthy non-obese volunteers without a history of the following: medication for at least 6 months prior to participation in the study, respiratory complications, hypertension, diabetes, or other chronic diseases).

### 2.3. Evaluation of Pulmonary Function

Pulmonary function was evaluated using forced spirometry (spirometer EasyOne^®^ Connect [3.9.0.19 (es)/PC-Sensor [2.0.0.0]). The spirometry was interpreted using the values of forced expiratory volume in the first second (FEV1) and forced vital capacity (FVC). Results were considered normal when the ratio FEV1/FVC was ≥70% and FVC was ≥80% of the predictive value. An abnormal spirometry was restrictive (FEV1/FVC was >70% and FVC was <80% of the predictive value) or obstructive (FEV1/FVC was <70% and FVC was >80% of the predictive value), using data from the third National Health and Nutrition Examination Survey (NHANES III) [18].

### 2.4. Blood Sample Collection and RNA Isolation

PBMCs were obtained from anticoagulated (EDTA) blood samples. The cells were isolated using Ficoll-Histopaque 1077 (Sigma-Aldrich, Darmstadt, Hesse, Germany) density gradient and stored at −80 °C until their use. Total RNA was isolated from PBMCs using TRIzol (Invitrogen, Waltham, MA, USA) according to the manufacturer’s instructions. Whole blood samples were centrifuged to obtain plasma samples, which were stored at −80 °C until their use.

### 2.5. Analysis of Relative Gene Expression

RNA was quantified using a Nanodrop 2000 spectrophotometer (ThermoFisher Scientific, Waltham, MA, USA), and its integrity was confirmed by agarose gel electrophoresis. RNA was reverse transcribed using the primer oligo (dT)18 included in the RevertAid H Minus First Strand cDNA Synthesis kit (ThermoFisher Scientific, Waltham, MA, USA). Reaction mixtures were performed using 1000 ng of total RNA and incubated at 65 °C for 5 min, 42 °C for 60 min, and 70 °C for 10 min.

The gene expression levels of IL-6, IL-12, IL-17, VEGF-A, and MIP-1A were determined using RT-PCR, FastStart Universal SYBR Green Master (Rox) (Roche, Risch, Zug, Switzerland) and 100 ng of cDNA. RNase P was used as endogenous expression control in all experiments. Due to RNA yields, not all gene expression levels could be determined in each post-COVID-19 syndrome patient. All reactions were run in duplicate using a MyGo Pro PCR instrument (MyGo PCR, Middlesbrough, UK) under the following conditions: 94 °C for 10 min, 40 cycles at 94 °C for 30 s, 60 °C for 30 s, and 72 °C for 30 s, with a final extension of 72 °C for 5 min. Prior to analysis of gene expression, the dynamic ranges and dissociation curves of each gene evaluated were calculated.

The primers used were as follows: IL-6 forward 5′-TAGAGTACCTCC ACAACAGA-3′, IL-6 reverse 5′-CACAAATCGCAGCCT GC-3′; IL-12 forward 5′ CCATGCCTTCACCACTCC 3′, IL-12 reverse 5′-TCAGAAGTTCAAGGGTAAAATTC-3′; IL-17 forward 5′-TCTCTTGCTGGATGGGGACA-3′, IL-17 reverse 5′-AACCGATCCACCTCACCTTG-3′; VEGF-A forward 5′-AGGGCAGAATCATCACGAAGT-3′, VEGF-A reverse 5′-AGGGTCTCGATTGGATGGCA-3′; MIP-1 forward 5′-TCCTGGTTCTTTTGCTGGTC-3′, MIP-1 reverse 5′-TCAGGGAAGGGATAGGGTTAGT-3′, RNaseP forward 5′-AGATTTGGACCTGCGAGCG-3′, and RNaseP reverse 5′-GAGCGGCGGCTGTCTCCACAAGT-3. The analysis of the relative gene expression was performed using the 2^−ΔΔCt^ method [19]. For the ΔCt of the reference sample, the average ΔCt for RNase P and each cytokine was calculated using the healthy control group.

### 2.6. MMP-9 Determination

Serum levels of MMP-9 were determined using the ELISA kit LEGEND MAX Human MMP-9 (BioLegend, San Diego, CA, USA), following the manufacturer’s instructions. The reading of the assay was performed in a spectrophotometer at 450 nm (Varioscan Lux; ThermoScientific, Waltham, MA, USA).

### 2.7. Statistical Analysis

The normality of the data was determined with the Shapiro–Wilk test. Differences between groups were evaluated with the student *t* test or the Mann–Whitney U test, based on data normality. Similarly, the data were expressed as the mean with standard deviation or the median with interquartile range. The statistical package GraphPad Prism version 10.1.1 was used. Categorical variables were expressed as frequencies and percentages. Pearson’s *X*^2^ or Fisher exact tests were used to determine associations. The analysis of risk was calculated via the odds ratio (OR) with a confidence interval of 95% (CI 95%) using the statistical package SPSS^®^ version 20 for Windows. A *p*-value ≤ 0.05 was considered significant.

## 3. Results

### 3.1. Instrument Evaluation, Participants’ Demographic Characteristics, and Clinical Parameters

A cohort of 50 subjects evaluated the instrument. The results indicate that the instrument is clear and comprehensible, obtaining scores of <2 (three-point scale) and ≤3 (ten-point scale). Regarding readability, the Flesch–Kincaid test showed an average score (INFLESZ software version 1.0) of 65.54 (scale from 0 to 100), showing that the instrument is easy to read.

A total 233 patients with post-COVID-19 syndrome diagnosis were contacted. Among these patients, 45 confirmed the persistence of symptoms and agreed to participate in the study (64% were female). Among the healthy control group (n = 5), three were female and the average age and body mass index (BMI) values were 33.4 ± 7.5 and 24.2 ± 0.54, respectively. The average age of the studied post-COVID-19 population was 46 ± 14 years, and the mean BMI was 29 ± 5.68. The comorbidities reported were obesity (47%), arterial hypertension (29%), diabetes mellitus (11%), and cardiomyopathic ischemia (2%). As many as 11% percent (n = 5) had not been COVID-19-vaccinated at the time of infection; 31% (n = 14) were hospitalized due to SARS-CoV-2 infection; and 33% (n = 15) were diagnosed with pneumonia associated with COVID-19. The most frequent persistent symptoms were fatigue (82%), dyspnea (53%), dry cough (47%), and muscle aches (22%) (Table 1).

### 3.2. Pulmonary Function and Comorbidities Associated with Pulmonary Alteration

Spirometry evaluations demonstrated that 62% (n = 28) of the patients had normal pulmonary function. Among those with abnormal spirometry (n = 17), 70.6% (n = 12) met criteria for a restrictive pattern, and 29.4% (n = 5) met the criteria for an obstructive respiratory pattern. Factors associated with the development of pulmonary alterations in patients with post-COVID-19 syndrome were pneumonia (OR: 6.5; CI 95%: 1.6 to 25.7; *p* = 0.005), history of hospitalization (OR: 5.1; CI 95%: 1.3 to 20.1; *p* = 0.01), severity of the illness (OR: 6.5; CI 95%: 1.6 to 25.7; *p* = 0.005), and the history of arterial hypertension (OR: 4.0; CI 95%: 1.05 to 15.8; *p* = 0.04) (Table 2). Furthermore, altered pulmonary function was associated with persistent symptoms of dyspnea (OR: 5.0; CI 95%: 1.29 to 19.4; *p* = 0.03), dry cough (OR: 3.3; CI 95%: 0.9 to 11.6; *p* = 0.05), and sleep disturbances (OR: 9.1; CI 95%: 1.6 to 51.4; *p* = 0.01) (Table 2). Although abnormal spirometry was not associated with the lack of vaccination before SARS-CoV-2 infection, most patients with normal spirometry were vaccinated before infection (75% (n = 21) vs. 25% (n = 7)). Furthermore, a lower percentage of vaccinated patients had abnormal spirometry (32% (n = 10) vs. 50% (n = 7)).

### 3.3. Inflammatory Cytokine Expression and Plasma Levels of MMP-9 Are Associated with Pulmonary Alteration

**Figure 1:**
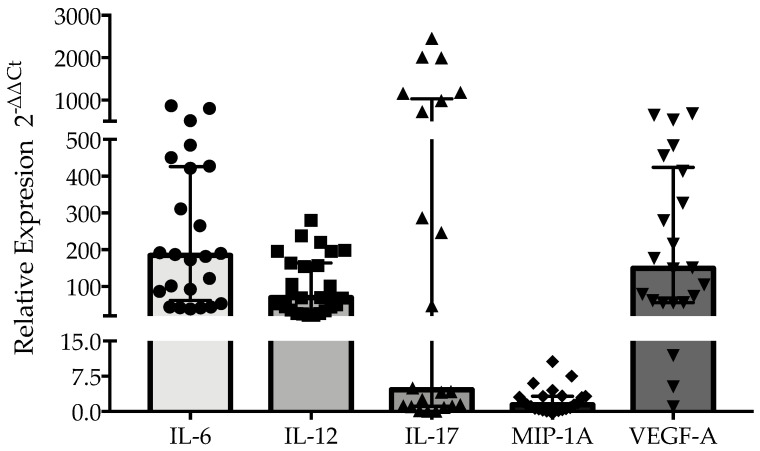
Relative gene expression of IL-6 (n = 25), IL-12 (n = 27), IL-17 (n = 22), MIP-1A (n = 23), and VEGF-A (n = 22) in the PBMCs of patients with post-COVID-19 syndrome. RNase P was used as the endogenous gene for gene expression analysis. Data are presented as the median and interquartile range.

**Figure 2:**
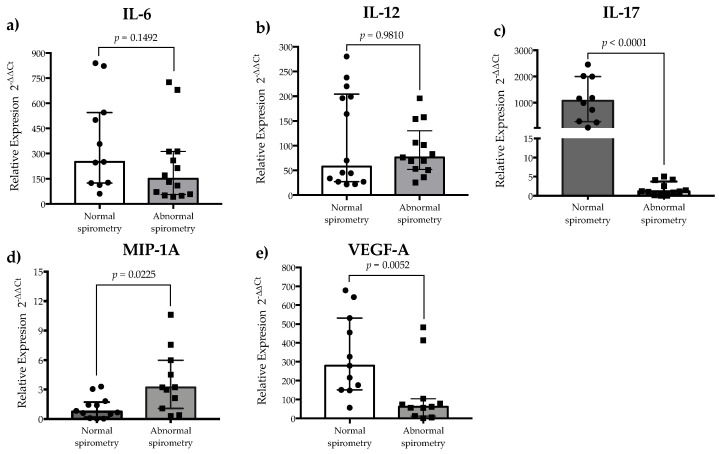
Relative gene expression of inflammatory cytokines and an angiogenic mediator in patients with post-COVID-19 syndrome with normal and abnormal spirometry: (**a**) IL-6; (**b**) IL-12; (**c**) IL-17; (**d**) MIP-1A; and (**e**) VEGF-A. RNase P was used as the endogenous gene for gene expression analysis. Data are presented as the median and interquartile range. Differences between groups for each biological marker were determined using the Mann–Whitney U test.

**Figure 3:**
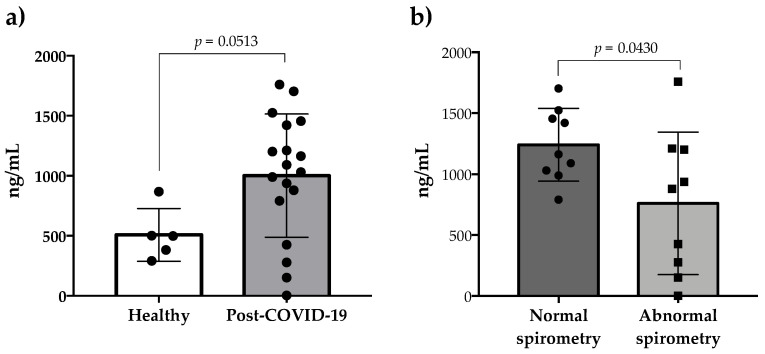
Plasma levels of MMP-9 in patients with post-COVID-19 syndrome with normal and abnormal spirometry. Data are presented as the mean and standard deviation. Difference between groups was determined using unpaired Student *t* test. In (**a**,**b**), each point represents a pool of two patients (except for one patient in the abnormal group).

The relative gene expressions of IL-17 and VEGF-A were highly variable in post-COVID-19 patients, but the expressions of IL-6 and IL-12 showed a trend of being upregulated, while the expression of MIP-1A remained at basal levels in 9 out of 23 patients evaluated (Figure 1). Comparisons between normal and abnormal spirometry groups showed that the relative gene expression of IL-6 and IL-12 were similar between the groups (*p* > 0.05) (Figure 2a and Figure 2b, respectively). On the contrary, the relative gene expressions of IL-17 and MIP-1A were significantly upregulated in the normal and abnormal spirometry patient groups, respectively (*p* < 0.05) (Figure 2c and Figure 2d, respectively). The relative gene expression of VEGF-A was significantly upregulated in the normal group (*p* < 0.05) (Figure 2e). Regarding MMP-9, the plasma levels of this metalloprotease were similar between post-COVID-19 patients and healthy controls (*p* > 0.05) but were higher in the normal spirometry group than in the abnormal one (*p* < 0.05) (Figure 3).

## 4. Discussion

In the present study, 45 patients with post-COVID-19 syndrome were evaluated for pulmonary function, inflammatory and angiogenic biomarkers, gene expression, and plasma levels of MMP-9. A questionnaire was designed to collect data about the persistence of COVID-19 symptoms, comorbidities, and vaccination, as well as demographic data. According to previous studies that established rigorous cut-off values for evaluating clarity, comprehension, and readability [15,16,17], the instrument developed was clear, comprehensible, and readable. Most patients were COVID-19-vaccinated with the first (95.5%) and second doses (84.4%), and many received a booster dose (68.9%). Thirty-one patients were vaccinated before SARS-CoV-2 infection. The proportion of patients with normal spirometry was up to three-fold higher in patients vaccinated before SARS-CoV-2 infection than in unvaccinated patients. Similarly, abnormal spirometry was more common in unvaccinated patients (70.6% vs. 29.4%) and in those with history of pneumonia, hypertension, and hospitalization. In this context, the results support the notion that COVID-19 vaccination before infection is associated with a reduced severity of post-COVID-19 syndrome [20], and that health systems should be aware of new SARS-CoV-2 variants and next-generation vaccines to reduce the severity of potential post-COVID-19 syndrome cases [21].

Although inflammatory cytokines and angiogenic factors are key mediators in the acute phase of SARS-CoV-2 infection [2], as well as in post-COVID-19 syndrome development [11], there is scarce evidence about their relationship with pulmonary function in this syndrome. Certainly, upregulation of IL-6 is associated with clinical complications during the acute phase of COVID-19, promoting pulmonary fibrosis in the resolution stage of SARS-CoV-2 infection [9,22]. Similarly, upregulation of IL-12 is associated with chronic inflammation and post-COVID-19 syndrome development [11]. In general, IL-6, IL-12, IL-17, and VEGF-A genes were upregulated in patients with post-COVID-19 syndrome compared to healthy subjects, but it should be acknowledged that the small size of the control group may limit the generalizability of these comparisons. Regarding spirometry results, the relative gene expressions of IL-6 and IL-12 were similar between the normal and abnormal spirometry groups, suggesting that these cytokines contribute to post-COVID-19 syndrome development but that they have limited value in predicting pulmonary function alterations in this syndrome.

It has been widely documented that IL-17 signaling could promote pulmonary function alterations in COVID-19 patients [23]. In fact, patients with active SARS-CoV-2 infection have increased IL-17 serum levels, and this has been associated with COVID-19 severity [24,25,26]. Others have reported an increased number of Th17 cells, the upregulation of IL-17 gene expression, and increased IL-17 serum levels in active COVID-19 patients compared with healthy subjects [27]. On the other hand, it has been documented that patients with post-COVID-19 syndrome, with or without signs of pulmonary sequelae radiologically, have lower IL-17 serum levels than healthy controls [28]. In line with these studies, the IL-17 gene expression data obtained in the present study show that IL-17 gene expression in PBMCs is upregulated in patients with post-COVID-19 syndrome compared to healthy controls. However, the relative gene expression of IL-17 was significantly higher in the group of patients with normal spirometry than those with abnormal spirometry. Paradoxically, these results suggest that upregulation of IL-17 gene expression in the PBMCs of patients with post-COVID-19 syndrome may be associated with adequate pulmonary function. A potential explanation for these findings could involve the ability of regulatory T cells to control the IL-17-dominated inflammatory response [27], but studies should be conducted to determine if there is an association between IL-17 gene expression and a reduced or raised regulatory T cell function in the PBMCs of post-COVID-19 patients with normal and abnormal spirometry.

Regarding MIP-1A, a variety of cells, such as macrophages, monocytes, lymphocytes, and fibroblasts, among others, can secrete this inflammatory and chemotactic chemokine upon stimulation with viral proteins, recruiting eosinophils, lymphocytes, and macrophages at inflammation sites [29]. In fact, the bronchoalveolar lavage fluid of patients with acute respiratory distress syndrome or those pulmonary fibrosis ones has increased MIP-1A levels [30,31]. In patients with active SARS-CoV-2 infection, serum/plasma levels of MIP-1A are increased, and peripheral blood monocytes have extensive vacuolization compared to healthy subjects, irrespective of severity [32]. Others have reported that plasma levels of MIP-1A are higher in ICU-admitted patients with COVID-19 than in those who did not require admission [33]. The present study evaluates the expression levels of MIP-1A in the PBMCs of patients with post-COVID-19 syndrome for the first time. The relative gene expression levels of this inflammatory chemokine were higher in the abnormal spirometry group than in the normal group, suggesting that the MIP-1A-mediated antiviral immunity triggered in COVID-19 patients that progress to post-COVID-19 syndrome with abnormal spirometry is more persistent than in post-COVID-19 syndrome cases with normal spirometry. Certainly, the serum levels of MIP-1A are significantly increased for up to five days after the first- and second-dose vaccinations [34]. In the present study, all the participants that were evaluated for MIP-1A gene expression received a second dose of COVID-19 vaccine before SARS-CoV-2 infection, but blood samples for the study were taken at least 6 months after COVID-19 diagnosis. Thus, the MIP-1A results are unlikely to be influenced by COVID-19 vaccination.

Although inflammation has been proposed as the main pathological pathway in COVID-19 patients, intussusceptive angiogenesis is an alternative pathological mechanism, mainly related to severe COVID-19 cases [35]. VEGF family members are central regulators of angiogenesis [36], and VEGF-A plays a pivotal role in orchestrating the abnormal growth of vessels in disease-related angiogenesis [37]. In this context, relative gene expression of VEGF-A is upregulated in the lung biopsies of patients who died from COVID-19 compared to the lung biopsies from donator controls [38], and the plasma levels of VEGF-A are higher in SARS-CoV-2-infected patients in the acute phase of the disease than in healthy subjects [33,39], highlighting the relevance of pathologic angiogenesis in active COVID-19 cases. Similarly, in the present study, the relative gene expression levels of VEGF-A in the PBMCs of patients with post-COVID-19 syndrome were increased compared to healthy controls. However, the post-COVID-19 syndrome group with abnormal spirometry showed lower gene expression levels of VEGF-A than the normal group. These results support the notion that VEGF-A may play a key role as a proangiogenic factor in the COVID-19 pathological pathway, but in post-COVID-19 syndrome, VEGF-A is regulated more efficiently in patients with abnormal spirometry than in those with normal pulmonary function. Exploring the potential mechanism underlying this biological behavior is beyond the scope of the present study. Certainly, this scientific challenge could include the evaluation of other angiogenic biological markers and even the determination of angiostatic ones.

Regarding MMP-9, this matrix metalloproteinase is an extracellular proteinase that can degrade extracellular matrix components and is involved in tissue remodeling, inflammation, and extracellular matrix biology [40]. Some studies have indicated that the plasma levels of MMP-9 are increased in active COVID-19 patients [41,42,43,44], as well as suggesting that this increase could be associated with COVID-19 mortality [41], and it could be an early indicator of respiratory failure [42,44]. In line with these studies, the plasma samples analyzed in the present study showed that the levels of MMP-9 were similar between patients with post-COVID-19 syndrome and healthy subjects, suggesting that this matrix metalloproteinase may have limited utility as a long-term biomarker for predicting respiratory failure. Between the normal and abnormal spirometry groups, the serum levels of MMP-9 were higher in the normal pulmonary function group than in the abnormal group. However, there was a tendency to match the MMP-9 levels between the groups. In this context, it should be noted that the blood samples from the post-COVID-19 patients enrolled in the present study were obtained at least 6 months after COVID-19 diagnosis, and that MMP-9 not only promotes the inflammation and degradation of lung tissue but also promotes alveolar epithelial repair [40].

## 5. Conclusions

The present study highlights that IL-6 and IL-12 gene expression levels are upregulated in the PBMCs of patients with post-COVID-19 syndrome compared to healthy controls, and that the relative gene expression levels of IL-17, VEGF-A, and MIP-1A might be potential prognostic predictors of post-COVID-19 syndrome, which is associated with pulmonary function. The results also highlight the complexity of the physiological and immune mechanisms underlying post-COVID-19 syndrome and set the groundwork for further research to contribute to our understanding of this syndrome.

## Figures and Tables

**Table 1:** Demographic characteristics and clinical parameters of post-COVID-19 patients.

Demographic Characteristics		n (%)
Sex	Male	16 (36)
	Female	29 (64)
Age (m ± SD)		46 ± 14.05
BMI (m ± SD)		29.37 ± 5.68
Comorbidities	Obesity	21 (47)
	Arterial hypertension	13 (29)
	Diabetes mellitus	5 (11)
	Cardiomyopathic ischemia	1 (2)
Substance abuse	Alcohol	21 (47)
	Tobacco	8 (18)
Type of vaccine administered	First dose (n = 43)	
	Pfizer	26 (58)
	Cansino	9 (20)
	Astra Zeneca	8 (18)
	Second dose (n = 38)	
	Pfizer	27 (60)
	Astra Zeneca	11 (24)
	Booster (n = 32)	
	Astra Zeneca	19 (42)
	Pfizer	12 (27)
	SINOVAC	1 (2)
Clinical Parameters		n (%)
Hospitalization		14 (31)
Diagnosis of pneumonia		15 (33)
Persistent symptoms	Fatigue	37 (82)
	Dyspnea	24 (53)
	Dry cough	21 (47)
	Muscle aches	10 (22)
	Sleep disturbances	9 (20)
	Joint pain	7 (16)
	Headache	7 (16)
	Palpitations	6 (13)
	Blurred vision	5 (11)
	Anosmia	4 (9)
	Dizziness	4 (9)

m = mean; SD = standard deviation.

**Table 2:** Variables associated with abnormal spirometry in patients with post-COVID-19 syndrome.

Variable	Spirometry		OR	CI 95%	*p*
	Abnormal (n = 17) n (%)	Normal (n = 28) n (%)			
Age over 60 years	5 (29.4)	3 (10.7)	3.4	0.7–16.9	0.22
Female	14 (82.4)	15 (53.6)	0.2	0.05–1.05	0.06
Obesity	9 (52.9)	12 (42.9)	1.5	0.4–5.0	0.51^+^
Pneumonia	10 (58.8)	5 (17.9)	6.5	1.6–25.7	0.005^+^
Hospitalization	9 (52.9)	5 (17.9)	5.1	1.3–20.1	0.014^+^
Severity (moderate- severe)	10 (58.8)	5 (17.9)	6.5	1.6–25.7	0.005^+^
No vaccine before the infection	7 (41.2)	7 (25)	2.1	0.5–7.6	0.25^+^
Alcohol	5 (29.4)	16 (57.1)	0.3	0.08–1.1	0.71^+^
Tobacco	4 (23.5)	4 (14.3)	1.8	0.3–8.6	0.45
Arterial hypertension	8 (47.1)	5 (17.9)	4.0	1.0–15.8	0.04
Diabetes mellitus	4 (23.5)	1 (3.6)	8.3	0.8–81.9	0.06
Cardiomyopathic ischemia	1 (5.9)	--	2.7	1.8–4.0	0.37
Persistence of symptoms					
Fatigue	14 (82.4)	23 (82.1)	1.0	0.2–4.9	1.0
Anosmia	1 (5.9)	3 (10.7)	0.5	0.05–5.4	1.0
Dyspnea	13 (76.5)	11 (39.3)	5.0	1.29–19.4	0.03
Dry cough	11 (64.7)	10 (35.7)	3.3	0.9–11.6	0.05
Muscle cramps	5 (29.4)	5 (17.9)	1.9	0.4–7.9	0.46
Joint pain	2 (11.8)	5 (17.9)	0.6	0.1–3.5	0.69
Sleep disturbances	7 (41.2)	2 (7.1)	9.1	1.6–51.4	0.01
Headache	3 (17.6)	4 (14.3)	1.2	0.25–6.5	1.0
Dizziness	1 (5.9)	3 (10.7)	0.5	0.05–5.4	1.0
Blurred vision	2 (11.8)	3 (10.7)	1.1	0.1–7.4	1.0
Palpitations	2 (11.8)	4 (14.3)	0.8	0.1–4.9	1.0

OR = odds ratio; CI 95% = confidence interval 95%; *p* = Fisher exact test; *p*^+^ = Pearson’s *X*^2^ test.

## Data Availability

The original contributions presented in this study are included in the article. Further inquiries can be directed to the corresponding author(s).

## Abbreviations

The following abbreviations are used in this manuscript:

BMI	Body mass index
CI 95%	Confidence interval of 95%
FEV1	Forced expiratory volume in the first second
FVC	Forced vital capacity
ICU	Intensive care unit
MIP	Macrophage inflammatory protein
MMP-9	Matrix metalloproteinase-9
OR	Odds ratio
PBMCs	Peripheral blood mononuclear cells
ROS	Reactive oxygen species
SARS-CoV-2	Severe acute respiratory syndrome-coronavirus-2
VEGF	Vascular endothelial growth factor
